# 

**Published:** 1875-01-15

**Authors:** 


*ORIGINAL COMMUNICATIONS:*

Biology. By N. S. Davis, M.D. .......... 27

Remarks upon Catalepsy, with Report of a Case. By J. H. Hollister, M.D. .......... 34

*CLINICAL REPORTS:*

Mercy Hospital Ophthalmic Clinic. By S. J. Jones, M.D. .......... 39

*TRANSLATIONS:*

Progress of Medical Science in Germany. By E. J. Doering, M.D. .......... 41

Gleanings from the French .......... 42

*EDITORIAL DEPARTMENT:*

Social Organization and Co-operation as a means of advancing Medical Science .......... 43

*SOCIETY REPORTS:*

Transactions of the Chicago Society of Physicians and Surgeons. Regular Meeting, Dec. 28, 1874 .......... 44

*GLEANINGS FROM OUR EXCHANGES:*

Report on Otology. By S. J. Jones, M.D. .......... 46

Nitrite of Amyl and Belladonna in Dysmenorrhœa .......... 49

*BOOK REVIEWS:* .......... 54

